# Can using the Cochrane RCT classifier in EPPI‐Reviewer help speed up study selection in qualitative evidence syntheses? A retrospective evaluation

**DOI:** 10.1002/cesm.70012

**Published:** 2025-01-13

**Authors:** Heather Melanie R. Ames, Christine Hillestad Hestevik, Patricia Sofia Jacobsen Jardim, Martin Smådal Larsen, Lars Jørun Langøien, Hans Bugge Bergsund, Tiril Cecilie Borge

**Affiliations:** ^1^ The Norwegian Institute of Public Health Oslo Norway

**Keywords:** artificial intelligence, classification, Cochrane RCT classifier, machine learning, qualitative evidence synthesis, systematic review automation

## Abstract

**Introduction:**

Using machine learning functions, such as study design classifiers, to automatically identify studies that do not meet the inclusion criteria, is one way to speed up the systematic review screening process. As a qualitative study design classifier is yet to be developed, using the Cochrane randomized controlled trial (RCT) classifier in reverse is one possible way to speed up the identification of primary qualitative studies during screening. The objective of this study was to evaluate whether the Cochrane RCT classifier can be used to speed up the study selection process for qualitative evidence synthesis (QES).

**Methods:**

We performed a retrospective evaluation where we first identified QES. We then extracted the bibliographic information of the included primary qualitative studies in each QES, and uploaded the references into our data management tool, EPPI‐Reviewer. We then ran the Cochrane RCT classifier on each group of included studies for each QES.

**Results:**

Eighty‐two QES with 2828 unique primary studies were included in the analysis. 56% of the primary studies were classified as unlikely to be an RCT and 40% as being 0–9% likely to be an RCT. 4% were classified as being 10% or more likely to be an RCT. Of these, only 1.7% were classified as being 50% or more likely to be an RCT.

**Conclusions:**

The Cochrane RCT classifier could be a useful tool to identify primary studies with qualitative study designs to speed up study selection in a QES. However, it is possible that mixed methods studies or qualitative studies conducted as part of a clinical trial may be missed. Further evaluations using the Cochrane RCT classifier on all the references retrieved from the complete literature search is needed to investigate time‐ and resource savings.

## 1 INTRODUCTION

Systematic reviews underpin evidence‐based policy and medicine. Many steps of the evidence synthesis process require highly trained reviewers and are complex but very repetitive. Machine learning (ML) tools can reduce the need for reviewers to conduct such repetitive and complex tasks.

Study selection is a particularly resource‐intensive step in the review process [1] and using ML to automatically determine irrelevance of studies that do not meet the inclusion criteria, without manual assessment, is one way to decrease the time used on study selection [2]. Classifiers are an application of supervised machine learning, in which a data set – here, the titles/abstracts and decisions that have been made by researchers, and whether a study shall be included or excluded — is used to train a model to make such decisions, thereby replacing human with machine effort. Several pretrained study design classifiers now exist for reviewers to use across reviews, perhaps most notably the Cochrane RCT classifier [3].

An increasing number of Norwegian Institute of Public Health (NIPH) reviews are reporting using binary ML classifiers (hereafter “classifiers”) to help in study selection [4, 5, 6, 7] such as the Cochrane randomized control trial (RCT) classifier. The Cochrane RCT classifier is recommended by the Cochrane Collaboration for systematic reviews of effect including only publications with an RCT study design. It is designed to categorize studies by their likelihood of being RCTs, that is, it gives a probability for each study (between 0% and 100%) that they are RCTs. (The output is shown in a bar chart, grouping studies into decile bands of probability – 0–9%, 10–19%, etc.). Studies with a low probability score (0.5 or less/50% or under) are unlikely to be RCTs. Those above 0.5 (or 50%) have a higher likelihood that they follow an RCT format. Lower scores suggest that the study is less likely to adhere to an RCT study design, often because it lacks terms or methodological markers associated with randomization and controlled trials. In practical terms, these studies are more likely to be observational, case reports, mixed methods or qualitative studies, or other non‐randomized designs.

The Cochrane RCT classifier was trained and tested with the help of Cochrane Crowd. The training set consisted of 280,620 title/abstract records from CENTRAL, published between 2014 and 2016. Cochrane Crowd categorized 7% of them as RCTs. The testing set consisted of 44,000 records from the Clinical Hedges data set. The Cochrane RCT classifier has over 99% recall and using it before starting the manual screening process is a recommended practice in Cochrane systematic reviews of effect [3].

Qualitative evidence syntheses (QES) apply principles of qualitative research to systematic reviews. A dedicated classifier for qualitative study designs does not yet exist. Creating one would require significant expertise, time, and resources. Most evidence synthesis groups do not have dedicated programmers to build customized tools and must rely on generic software. In the meantime, using the Cochrane RCT classifier in reverse to identify studies unlikely to be RCTs is one possible way to speed up the identification of primary qualitative studies during the title and abstract screening process.

QES can be used in guideline processes to provide information on acceptability, feasibility, experiences of an intervention and questions related to equity [8, 9, 10]. QES are often conducted in an iterative cyclical process with adjustments to inclusion criteria along the way. Various methods for sampling for included studies may also occur [11]. Practically, this also means that expectations of the study selection process are different from that of a review of effect, as a QES does not need to identify every study that meets inclusion criteria. QES are looking to include a broad spectrum of studies on the topic of interest that cover different perspectives and experiences to give a whole picture, not the comprehensive data set of all studies published on a topic of interest. Nevertheless, QES typically require reviewers to read and assess many studies for eligibility, some of which use ambiguous language, unclear titles and abstracts and descriptions of study design that make them hard to screen efficiently. The semi‐ or full automation of this step could likely result in significant resource savings. However, to our knowledge, classifiers have not been tested or evaluated on QES. Hence, the objective of this study was to evaluate whether the Cochrane RCT classifier can be used to speed up the study selection process for QES.

## 2 MATERIALS AND METHODS

This retrospective evaluation has been conducted by the machine learning team at the Norwegian Institute of Public Health. We have been working with how to best implement ML in systematic reviews based on the tools in EPPI‐Reviewer since late 2020. We have recently published an implementation guidance for other evidence synthesis groups that want to implement machine learning in their evidence synthesis workflow [12].

Our approach for this retrospective evaluation consisted of four steps:

1) Identify QES;
2) Determine the included primary studies in each QES;
3) Find and upload the appropriate reference data to the identified studies into EPPI‐Reviewer; and
4) Run the Cochrane RCT classifier on (a) each group of primary studies per QES, and (b) a single group consisting of all included primary studies.

### 2.1 Identify QES

We identified QES to be included in this project in four ways: First, we used all reviews labeled as QES in the Cochrane library as of July 12, 2023. Second, we sent an email out to our research networks asking for authors to submit RIS files to us from any QES they had authored where screening was completed. Thirdly, we identified QES while screening for other systematic reviews or by searching in Google Scholar. Finally, we extracted the QES included in overviews of QES we identified through the above steps. These QES were compiled into a single list in a word document for the article authors to work from.

### 2.2 Determine the included primary studies in each QES

We divided the list of identified QES between the article authors and created a comprehensive methods document providing step‐by‐step instructions for the project. Each author found the full text of the QES assigned to them and determined the number of included studies if we had access to the full text. If no full text access was available this was noted. Subsequently, the author determined the number of included studies in the review, utilizing either the table of included studies or the findings section (if no table was available) to select the references for export. We chose to include all studies meeting the inclusion criteria if possible, in QES where study sampling into the analysis was used.

### 2.3 Find and upload the appropriate reference data to the identified studies into EPPI‐Reviewer

Each author then located the QES in Web of Science (WOS) and the relevant references were exported to an EndNote [13] file for the QES. If a reference was not available in WOS, authors searched for it in Google Scholar and identified the included primary studies through individual searches. It was not always feasible to find references for all included studies; in some cases, only references for sampled studies were available, omitting some studies that met the inclusion criteria. In some instances, QES authors provided a RIS file of their included studies for review.

After the references were imported to EndNote, any missing abstracts were identified and added. The file was then exported and uploaded into EPPI‐Reviewer [14]. A code was created in EPPI‐Reviewer for each QES, and the references assigned appropriately.

### 2.4 Run the RCT classifier on each group of primary studies per QES

**Figure 1:**
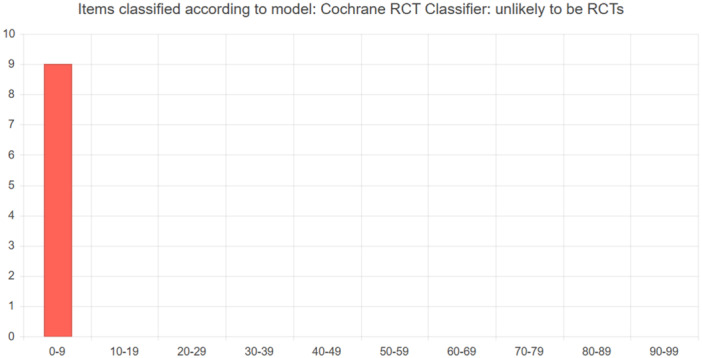
An example of distribution of items classified by the Cochrane RCT classifier as unlikely to be RCTS.

Having uploaded the references into EPPI‐Reviewer, we ran the Cochrane RCT classifier [3] on each group of included studies for each QES. The output from running the Cochrane RCT classifier is scores shown as integers between 0 and 99, corresponding to predicted probability of being an RCT or not. The cutoff threshold to obtain 99% recall, that is, to be placed in the “may be an RCT”, is 0.24%. Thus, all items with 0.24% or less in their score end in the “unlikely to be an RCT” group, and they all appear in the 0–9 range, because they have less than 1% probability of being an RCT (Figure 1). Correspondingly, items with “0” as their score in the “may be an RCT” group have a score which is more or equal to 0.24 [3].

### 2.5 Run the RCT classifier on a single group consisting of all included primary studies

After the classifier had been applied, we coded the studies within each probability score to a separate code set and recorded them in an excel spread sheet.

After uploading and running each of the QES individually, the primary studies were deduplicated. The deduplicated data set was run as a single group of studies through the Cochrane RCT classifier again.

## 3 RESULTS

**Figure 2:**
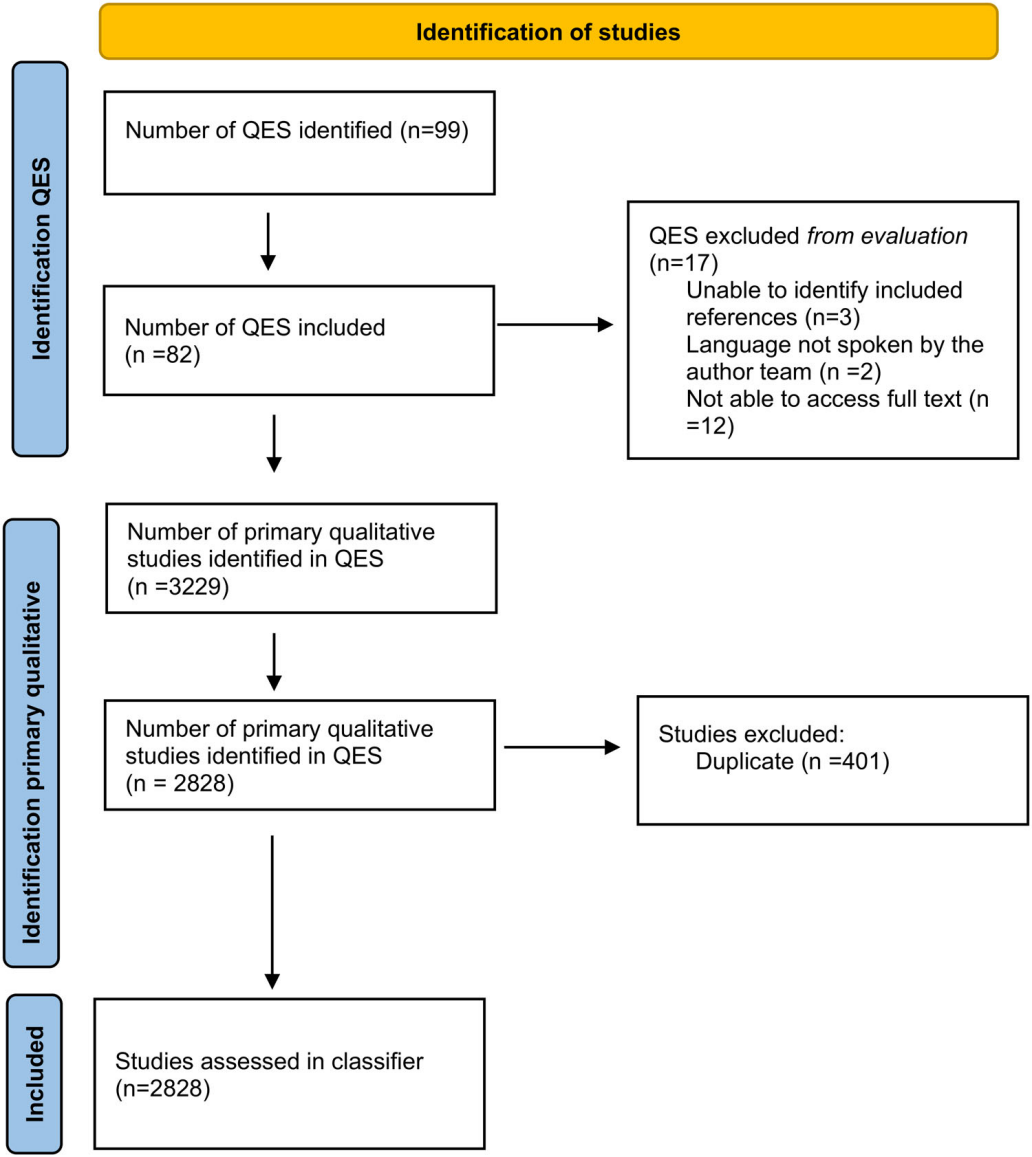
Flow diagram of the study identification process.

A total of 99 QES were identified. Seventeen were excluded from the analysis for the following reasons; Unable to identify the references for the included studies (*n* = 3) [15, 16, 17], language not spoken by the author team (*n* = 2; Persian [18]/Japanese [19]) and unable to access full text (*n* = 12) [20, 21, 22, 23, 24, 25, 26, 27, 28, 29, 30, 31]. Eighty‐two QES [4, 32, 33, 34, 35, 36, 37, 38, 39, 40, 41, 42, 43, 44, 45, 46, 47, 48, 49, 50, 51, 52, 53, 54, 55, 56, 57, 58, 59, 60, 61, 62, 63, 64, 65, 66, 67, 68, 69, 70, 71, 72, 73, 74, 75, 76, 77, 78, 79, 80, 81, 82, 83, 84, 85, 86, 87, 88, 89, 90, 91, 92, 93, 94, 95, 96, 97, 98, 99, 100, 101, 102, 103, 104, 105, 106, 107, 108, 109, 110, 111, 112] were included in the analysis with a total of 3229 primary studies before deduplication. After deduplication there were 2828 primary qualitative studies remaining (Figure 2). We could not find abstracts for 31 of the primary studies included in the evaluation.

For results per QES before deduplication see supplementary information file 1.

**Figure 3:**
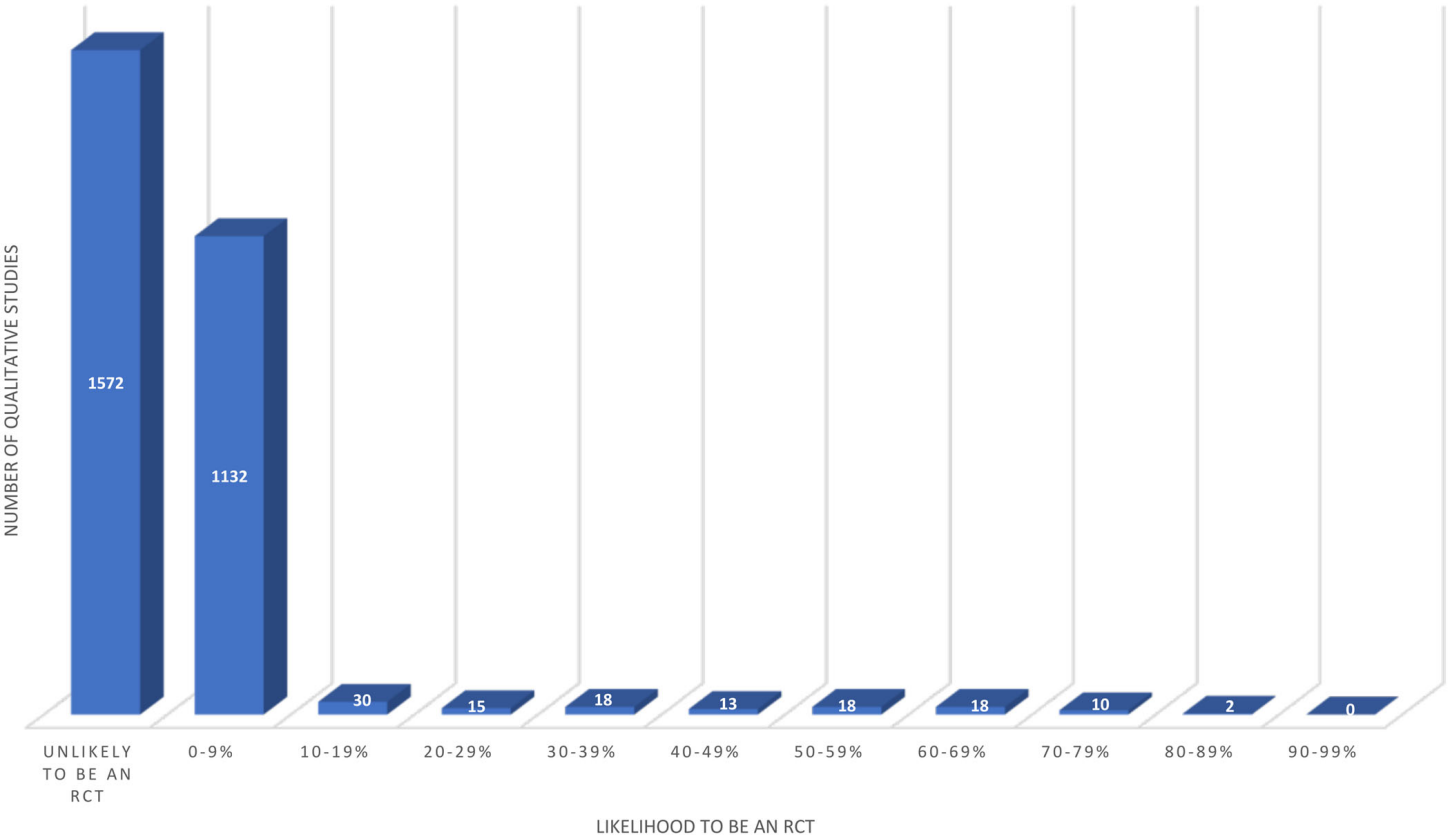
Likelihood that a primary qualitative study included in a QES was an RCT.

In this section we will present the results of the deduplicated findings. Most of the included primary studies (96%/*n* = 2704) were classified as unlikely to be an RCT (56%/*n* = 1572) or as being 0–9% likely to be an RCT (40%/*n* = 1132). The remaining studies (4%/*n* = 124) were classified as being 10% or more likely to be an RCT as shown in Figure 3.

**Figure 4:**
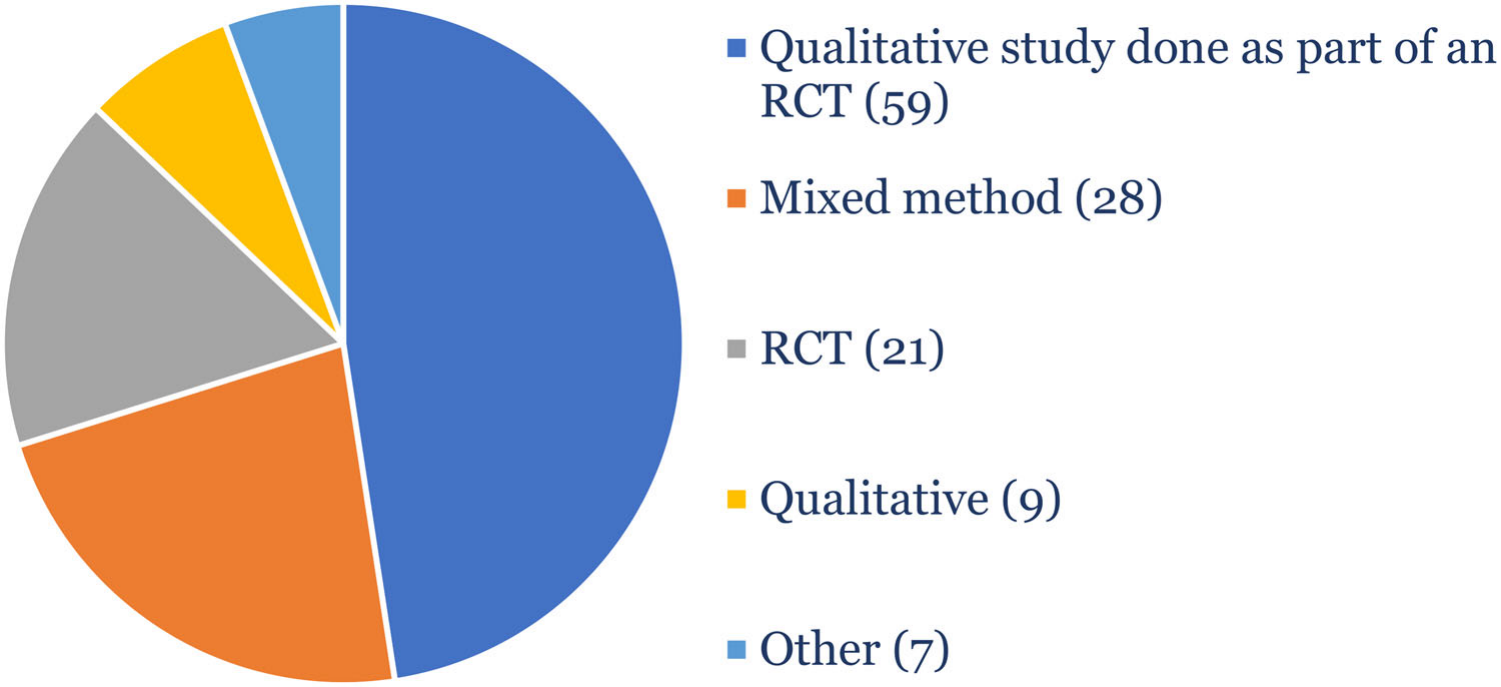
Study designs of the qualitative studies (*N* = 124) classified as being 10% or more likely to be an RCT.

We found that only 4.4% (*n* = 124) of the included studies were classified as being 10% or more likely to be an RCT. Twenty‐one (17%) of these were correctly classified as RCTs. These were included in the QES as they were sister studies of qualitative studies done during the trial. Only 7% (*n* = 9) were standalone qualitative studies. Forty‐eight percent (*n* = 59) were qualitative studies conducted as part of a trial (Figure 4).

**Figure 5:**
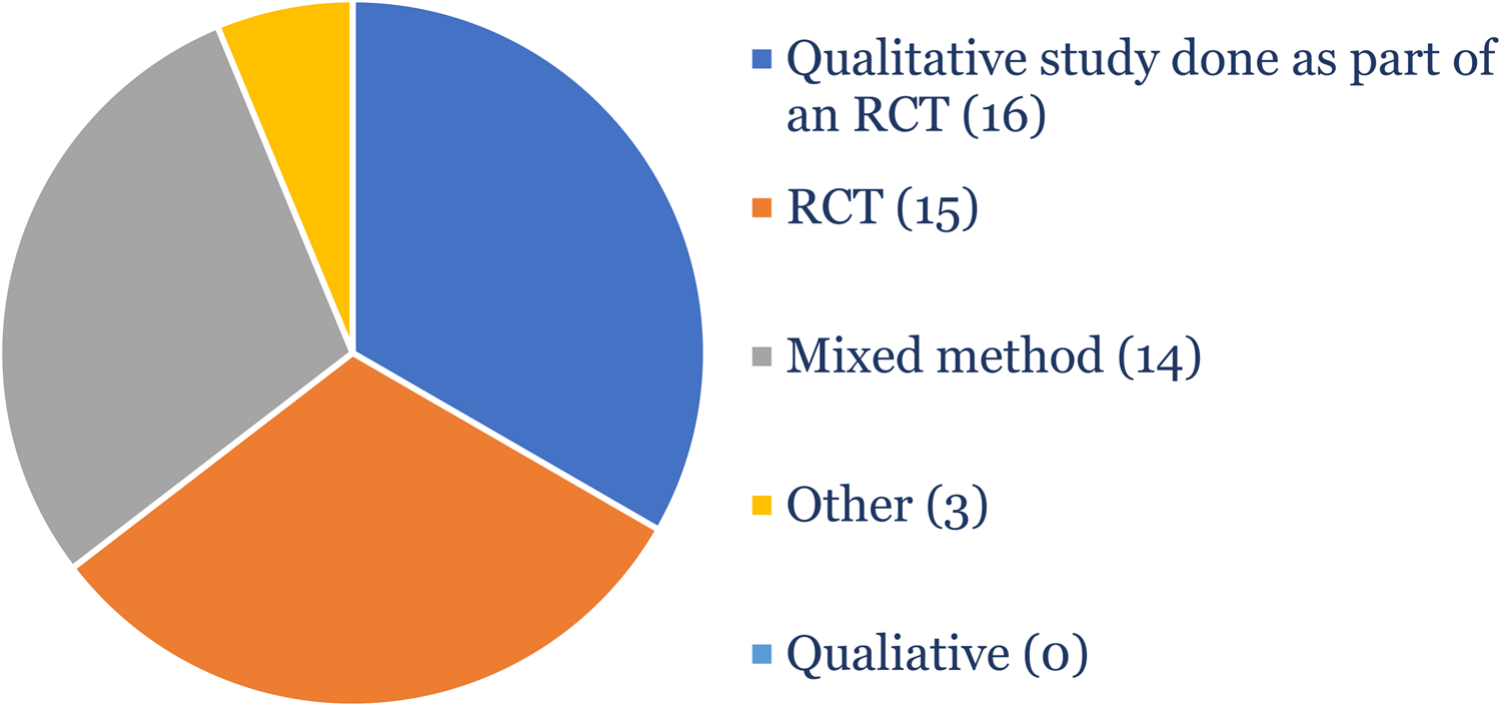
Study designs of the studies (*N* = 48) classified as being 50% or more likely to be an RCT.

Only 1.7% (*n* = 48) of the included studies were 50% or more likely to be an RCT. Of these 15 (31%) were correctly identified as RCTs. None of these studies were a standalone qualitative study. Sixteen (33%) of these studies were qualitative studies conducted as part of a trial where the trial or trial registry number was mentioned in the abstract and/or title (Figure 5).

## 4 DISCUSSION

**Figure 6:**
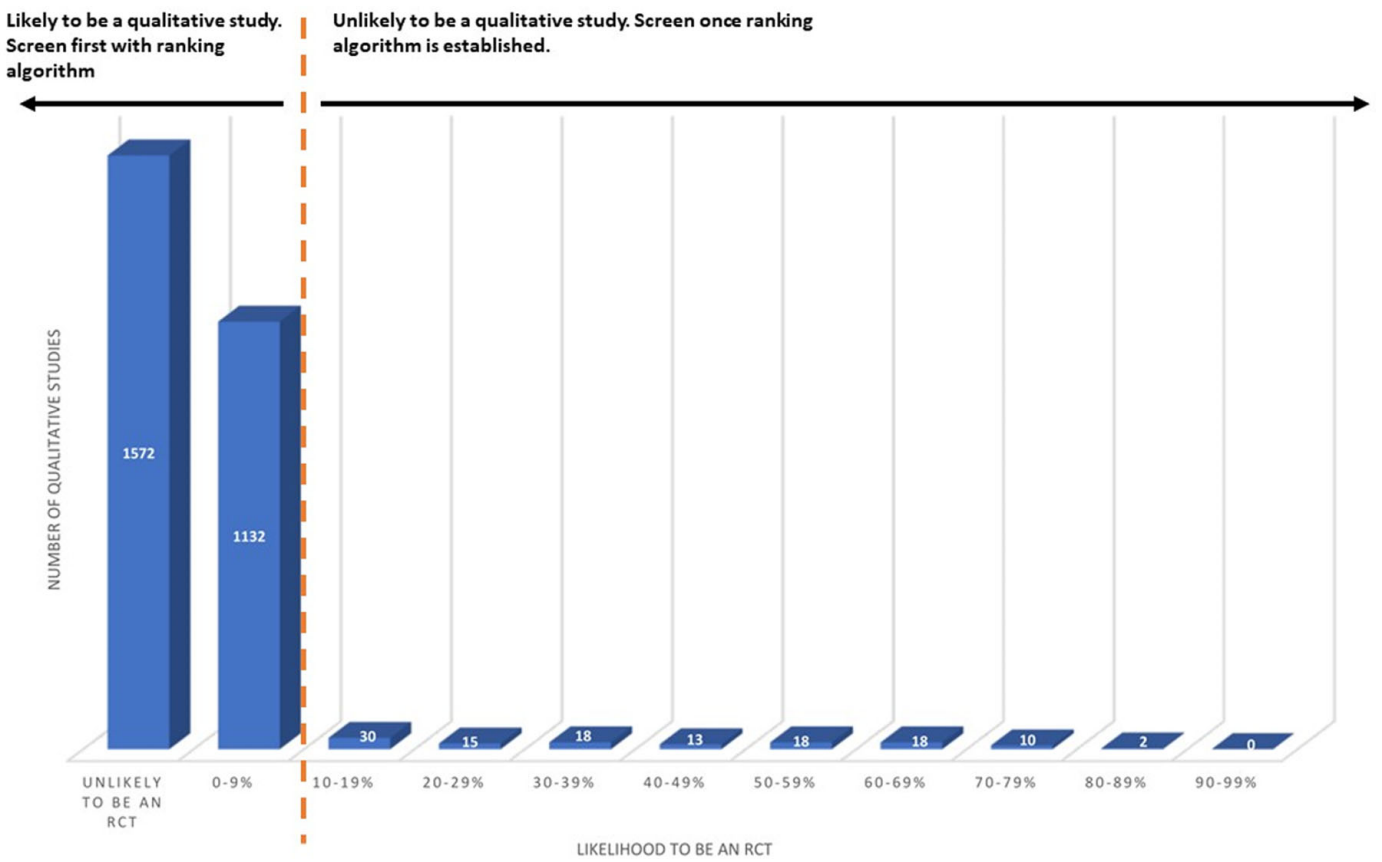
An example of using the 10% cutoff point and screening with a rank‐ing algorithm.

This retrospective evaluation found that by using the Cochrane RCT classifier it is possible to identify the majority of relevant primary studies using qualitative methods, especially if authors choose to begin with a 10% cutoff point. By a 10% cutoff point we mean that all studies classified in the 10–19% likely to be an RCT and higher would not be screened or would be included in screening with a ranking algorithm, called priority screening in EPPI‐Reviewer, once one has been established. However, it is possible that mixed methods studies or qualitative studies conducted as part of a trial may be classified as having a higher probability of being an RCT and would not be screened if a fixed cutoff threshold was being implemented. See Figure 6 for a visual example.

Authors will need to make an independent decision based on the topic of their review and the likelihood that qualitative studies linked to trials exist when deciding if they are going to automatically exclude studies over a certain probability of being an RCT. For example, a QES on a medicalized topic such as experiences with a certain medication, treatment or diagnosis is more likely to have qualitative studies linked to trials or mixed methods studies than a QES on a person's experience living with a disease or an informal intervention in the health care system. Furthermore, qualitative studies embedded in RCTs, mixed method studies, and studies using quantitative terms when describing the study like pilot, intervention, trial, evaluation, comparison, controlled etc., are more likely to be classified as an RCT than studies using qualitative terminology. Further research is needed to see if these studies would have been picked up and included through other methods such as reference checking or citation chasing.

The findings of this evaluation are a step toward how primary qualitative studies can be identified more quickly in a set of references retrieved from a literature search. However, this approach is best used in conjunction with other machine learning functions, such as ranking algorithms. When applying this approach in our QES we have started screening the studies unlikely to be RCTs first using a ranking algorithm. Once the ranking algorithm is identifying studies that meet our inclusion criteria, we have added the studies that are likely to be an RCT to the pool of studies to be screened. In this way, we have avoided screening a lot of studies with irrelevant study designs. We have adopted this approach because QES literature searches have often already used a qualitative filter in the literature search process so the time savings for auto exclusion may be small.

Further research is needed to confirm the results of our evaluation. Further expansion and a systematic QES identification method to ensure representation from different research areas would allow us to see if the classification pattern we have found for health‐related QES is like those in other areas. Further research would also potentially allow for identifying a cutoff point for auto exclusion or if studies above a certain likelihood of being an RCT would have been identified through reference checking or citation chasing. Also, to be able to estimate time‐ and resource savings, evaluations using the Cochrane RCT classifier on all the references retrieved from the complete literature search will have to be conducted.

While conducting this evaluation we identified some issues linked to the transparency of reporting of primary qualitative studies included in a QES. Three QES had no transparent reporting of the studies included in the synthesis or references in the findings section so these could not be included. Transparency of reporting varies across the 82 QES we included in the analysis. Some have tables which list all the included primary studies. This was the most transparent way to identify included primary studies. Some gave a total number at the beginning of the findings section but did not provide a table or references, and some gave no total number but referenced studies in the findings section. For QES using the last two approaches, it was difficult and much more time consuming to identify which studies had been used in the synthesis.

We also identified variations as to what QES authors list as an included study. This variation has contributed to the variation in the results. For example, 21 of the included studies were RCTs and correctly identified by the Cochrane RCT classifier.

We recognize that this study has some weaknesses. We did not systematically search for and select QES. This means that the representation of QES from different fields of research may be lacking, and we recognize an overrepresentation of health‐related topics. We have also not conducted an advanced analysis of the results of our evaluation. A different approach such as a receiver operating characteristic (ROC) analysis, may have given more insight into the changes in accuracy of using different cutoff points. Also, as we only used qualitative studies included at full text level for each QES, rather than the whole literature search results for each QES, we are unable to say anything about possible time‐ and resource savings throughout the whole screening process.

As we took this methodological approach, we were unable to see how many studies from the original search results would have fallen above the 10% threshold. Time savings for the author teams would be linked to this number as well as the total number of search hits for the QES. A larger number of studies to screen would potentially increase time‐ and resource savings when compared to a QES with a limited number of studies to screen. Time‐ and resource‐savings could also be linked to other machine learning tools used by the author team such as a ranking algorithm, other classifiers or automatic text clustering.

## 5 CONCLUSIONS

This retrospective evaluation determined that the Cochrane RCT classifier used in reverse allows qualitative primary studies to be identified, particularly when a 10% cutoff point is applied for initial screening. However, it is possible that mixed methods studies or qualitative studies conducted as part of a trial may be missed. Ideally, authors would begin screening studies unlikely to be an RCT first, in conjunction with using a ranking algorithm, and then expand to screen the remaining studies more likely to be RCTs after to ensure the identification of relevant studies classified as 10% or more likely to be an RCT. Further evaluations using the Cochrane RCT classifier on all the references retrieved from the complete literature search are needed to investigate time‐ and resource savings.

## AUTHOR CONTRIBUTIONS

**Heather Melanie R Ames**: Conceptualization; Data curation; Formal analysis; Methodology; Project administration; Visualization; Writing—original draft; Writing—review and editing. **Christine Hillestad Hestevik**: Conceptualization; Data curation; Methodology; Visualization; Writing—original draft; Writing—review and editing. **Patricia Sofia Jacobsen Jardim**: Conceptualization; Data curation; Methodology; Visualization; Writing—original draft; Writing—review and editing. **Martin Smådal Larsen**: Conceptualization; Data curation; Methodology; Writing—review and editing. **Lars Jørun Langøien**: Conceptualization; Data curation; Methodology; Writing—original draft; Writing—review and editing. **Hans Bugge Bergsund**: Conceptualization; Data curation; Methodology; Writing—original draft; Writing—review and editing. **Tiril Cecilie Borge**: Conceptualization; Data curation; Methodology; Project administration; Writing—original draft; Writing—review and editing.

## CONFLICT OF INTEREST STATEMENT

The authors declare no conflicts of interest.

## PEER REVIEW

The peer review history for this article is available at https://www.webofscience.com/api/gateway/wos/peer-review/10.1002/cesm.70012.

## Supporting information

Supporting information.

## Data Availability

All of the data included in this evaluation is available from the authors upon request.
