# Supplementary material for: Can using the Cochrane RCT classifier in EPPI‐Reviewer help speed up study selection in qualitative evidence syntheses? A retrospective evaluation
Source: Cochrane Evid Synth Methods. 2025 Jan 13;3(1):e70012. doi: 10.1002/cesm.70012 (PMC11795947; doi:10.1002/cesm.70012)
Supplement: Supplementary file 1 — Supporting information. [file CESM-3-e70012-s001.docx]

Supplementary information 1: Detailed table of classification by QES

|  | **Reference** | **Total N** | **Unlikely to be an RCT** | **0-9%** | **10-19%** | **20-29%** | **30-39%** | **40-49%** | **50-59%** | **60-69%** | **70-79%** | **80-89%** | **90-99%** |
| --- | --- | --- | --- | --- | --- | --- | --- | --- | --- | --- | --- | --- | --- |
| 1 | Afferri, A., Allen, H., Booth, A., Dierickx, S., Pacey, A., & Balen, J. (2022). Barriers and facilitators for the inclusion of fertility care in reproductive health policies in Africa: a qualitative evidence synthesis. Human reproduction update, 28(2), 190-199. | 20 | 20 |  |  |  |  |  |  |  |  |  |  |
| 2 | Agyenim-Boateng, A., Cameron, H., & Mensah, A. B. B. (2021). Health professionals’ perception of disrespectful and abusive intrapartum care during facility-based childbirth in LMIC: a qualitative systematic review and thematic synthesis. *International Journal of Africa Nursing Sciences*, *15*, 100326. | 13 | 11 | 2 |  |  |  |  |  |  |  |  |  |
| 3 | Akther, S. F., Molyneaux, E., Stuart, R., Johnson, S., Simpson, A., & Oram, S. (2019). Patients' experiences of assessment and detention under mental health legislation: systematic review and qualitative meta-synthesis. *BJPsych Open*, *5*(3), e37. | 55 | 41 | 14 |  |  |  |  |  |  |  |  |  |
| 4 | Allison, R. J., & Flemming, K. A. (2019). Mental health patients’ experiences of softer coercion and its effects on their interactions with practitioners: A qualitative evidence synthesis. *Journal of advanced nursing*. | 11 | 7 | 4 |  |  |  |  |  |  |  |  |  |
| 5 | Alves, S., Martins, A., Fonseca, A., Canavarro, M. C., & Pereira, M. (2018). Preventing and treating women’s postpartum depression: a qualitative systematic review on partner-inclusive interventions. *Journal of Child and Family Studies*, *27*, 1-25. | 14 |  | 1 | 3 |  | 2 | 3 | 1 | 1 | 2 | 1 |  |
| 6 | Ames, H., Hestevik, C. H., & Briggs, A. M. (2024). Acceptability, values, and preferences of older people for chronic low back pain management; a qualitative evidence synthesis. *BMC Geriatrics*, *24*(1), 24. https://doi.org/10.1186/s12877-023-04608-4 | 24 | 5 | 16 |  | 1 |  |  | 2 |  |  |  |  |
| 7 | Ames, H., Mosdøl, A., Blaasvær, N., Nøkleby, H., Berg, R., & Langøien, L. (2020). Communication of children’s weight status: what is effective and what are the children’s and parents’ experiences and preferences? A mixed methods systematic review. *BMC Public Health*, *20*(1), 1-22. | 25 | 9 | 15 |  |  |  | 1 |  |  |  |  |  |
| 8 | Ames, H. M., Glenton, C., & Lewin, S. (2017). Parents' and informal caregivers' views and experiences of communication about routine childhood vaccination: a synthesis of qualitative evidence. *Cochrane Database of Systematic Reviews*(2). | 75 | 46 | 28 |  |  | 1 |  |  |  |  |  |  |
| 9 | Ames, H. M., Glenton, C., Lewin, S., Tamrat, T., Akama, E., Leon, N., Consumers, C., & Group, C. (2019). Clients’ perceptions and experiences of targeted digital communication accessible via mobile devices for reproductive, maternal, newborn, child, and adolescent health: a qualitative evidence synthesis. *Cochrane Database of Systematic Reviews*, *2019*(10). | 50 | 10 | 30 | 2 | 1 | 2 | 3 | 2 |  |  |  |  |
| 10 | Andersen, M. F., Nielsen, K. M., & Brinkmann, S. (2012). Meta-synthesis of qualitative research on return to work among employees with common mental disorders. *Scandinavian journal of work, environment & health*, 93-104. | 8 | 7 | 1 |  |  |  |  |  |  |  |  |  |
| 11 | Bashah, D. T., Worku, A. G., & Mengistu, M. Y. (2018). Consequences of obstetric fistula in sub Sahara African countries, from patients’ perspective: a systematic review of qualitative studies. *BMC women's health*, *18*(1), 1-12. | 15 | 5 | 10 |  |  |  |  |  |  |  |  |  |
| 12 | Bohren, M. A., Berger, B. O., Munthe‐Kaas, H., & Tunçalp, Ö. (2019). Perceptions and experiences of labour companionship: a qualitative evidence synthesis. *Cochrane Database of Systematic Reviews*(3). | 52 | 37 | 15 |  |  |  |  |  |  |  |  |  |
| 13 | Breeksema, J. J., Niemeijer, A. R., Krediet, E., Vermetten, E., & Schoevers, R. A. (2020). Psychedelic treatments for psychiatric disorders: A systematic review and thematic synthesis of patient experiences in qualitative studies. *CNS drugs*, *34*, 925-946. | 15 | 6 | 9 |  |  |  |  |  |  |  |  |  |
| 14 | Brown, A., & O’Connor, S. (2020). Mobile health applications for people with dementia: a systematic review and synthesis of qualitative studies. *Informatics for Health and Social Care*, *45*(4), 343-359. | 9 | 3 | 6 |  |  |  |  |  |  |  |  |  |
| 15 | Brown, S. J., Carter, G. J., Halliwell, G., Brown, K., Caswell, R., Howarth, E., Feder, G., & O'Doherty, L. (2022). Survivor, family and professional experiences of psychosocial interventions for sexual abuse and violence: a qualitative evidence synthesis. *Cochrane Database of Systematic Reviews*(10). | 45 | 52 | 2 | 1 | 1 |  | 2 | 2 |  |  |  |  |
| 16 | Bunzli, S., Watkins, R., Smith, A., Schütze, R., & O’Sullivan, P. (2013). Lives on hold: a qualitative synthesis exploring the experience of chronic low-back pain. *The Clinical journal of pain*, *29*(10), 907-916. | 25 | 12 | 13 |  |  |  |  |  |  |  |  |  |
| 17 | Byrne, R., Davies, L., & Morrison, A. P. (2010). Priorities and preferences for the outcomes of treatment of psychosis: a service user perspective. *Psychosis*, *2*(3), 210-217. | 8 | 5 | 2 |  |  | 1 |  |  |  |  |  |  |
| 18 | Campbell, K., Coleman-Haynes, T., Bowker, K., Cooper, S. E., Connelly, S., & Coleman, T. (2020). Factors influencing the uptake and use of nicotine replacement therapy and e‐cigarettes in pregnant women who smoke: a qualitative evidence synthesis. *Cochrane Database of Systematic Reviews*, *2020*(5). | 21 | 8 | 11 | 1 |  | 1 |  |  |  |  |  |  |
| 19 | Carroll, C., Booth, A., Campbell, F., & Relton, C. (2020). What are the implications of Zika Virus for infant feeding? A synthesis of qualitative evidence concerning Congenital Zika Syndrome (CZS) and comparable conditions. *PLoS neglected tropical diseases*, *14*(10), e0008731. | 14 | 8 | 5 | 1 |  |  |  |  |  |  |  |  |
| 20 | Cleary, M., West, S., Hunt, G. E., McLean, L., & Kornhaber, R. (2020). A qualitative systematic review of caregivers’ experiences of caring for family diagnosed with schizophrenia. *Issues in mental health nursing*, *41*(8), 667-683. | 43 | 36 | 7 |  |  |  |  |  |  |  |  |  |
| 21 | Cooke, E., Smith, V., & Brenner, M. (2020). Parents’ experiences of accessing respite care for children with Autism Spectrum Disorder (ASD) at the acute and primary care interface: A systematic review. *BMC pediatrics*, *20*, 1-12. | 7 | 5 | 2 |  |  |  |  |  |  |  |  |  |
| 22 | Cooper, S., Schmidt, B.-M., Sambala, E. Z., Swartz, A., Colvin, C. J., Leon, N., & Wiysonge, C. S. (2021). Factors that influence parents' and informal caregivers' views and practices regarding routine childhood vaccination: a qualitative evidence synthesis. *Cochrane Database of Systematic Reviews*(10). | 176 | 126 | 50 |  |  |  |  |  |  |  |  |  |
| 23 | Cooper, S., Schmidt, B. M., Ryan, J., Leon, N., Mavundza, E., Burnett, R., Tanywe, A. C., & Wiysonge, C. S. (2019). Factors that influence acceptance of human papillomavirus (HPV) vaccination for adolescents: a qualitative evidence synthesis. *The Cochrane Database of Systematic Reviews*, *2019*(9). *Protocol-review not yet published. Data set received from the author. | 239 | 107 | 128 | 3 | 1 |  |  |  |  |  |  |  |
| 24 | Corring, D., O'Reilly, R. L., Sommerdyk, C., & Russell, E. (2018). What clinicians say about the experience of working with individuals on community treatment orders. *Psychiatric Services*, *69*(7), 791-796. | 12 | 10 | 2 |  |  |  |  |  |  |  |  |  |
| 25 | Dawson, S., Lawn, S., Simpson, A., & Muir-Cochrane, E. (2016). Care planning for consumers on community treatment orders: an integrative literature review. *BMC psychiatry*, *16*(1), 1-14. | 24 | 19 | 5 |  |  |  |  |  |  |  |  |  |
| 26 | de Waardt, D. A., van Melle, A. L., Widdershoven, G. A. M., Bramer, W. M., van der Heijden, F. M. M. A., Rugkåsa, J., & Mulder, C. L. (2022). Use of compulsory community treatment in mental healthcare: an integrative review of stakeholders’ opinions. *Frontiers in Psychiatry*, *13*, 1011961. | 22 | 18 | 4 |  |  |  |  |  |  |  |  |  |
| 27 | Doedens, P., Vermeulen, J., Boyette, L. L., Latour, C., & de Haan, L. (2020). Influence of nursing staff attitudes and characteristics on the use of coercive measures in acute mental health services—A systematic review. *Journal of Psychiatric and Mental Health Nursing*, *27*(4), 446-459. | 22 | 18 | 4 |  |  |  |  |  |  |  |  |  |
| 28 | Downe, S., Finlayson, K., Tunçalp, Ö., & Gülmezoglu, A. M. (2019). Provision and uptake of routine antenatal services: a qualitative evidence synthesis. *Cochrane Database of Systematic Reviews*(6). | 85 | 58 | 26 |  |  |  |  | 1 |  |  |  |  |
| 29 | Engel, N., Ochodo, E. A., Karanja, P. W., Schmidt, B.-M., Janssen, R., Steingart, K. R., & Oliver, S. (2022). Rapid molecular tests for tuberculosis and tuberculosis drug resistance: a qualitative evidence synthesis of recipient and provider views. *Cochrane Database of Systematic Reviews*(4). | 32 | 19 | 12 |  | 1 |  |  |  |  |  |  |  |
| 30 | Esteban, E., Coenen, M., Ito, E., Gruber, S., Scaratti, C., Leonardi, M., Roka, O., Vasilou, E., Muñoz-Murillo, A., & Ávila, C. C. (2018). Views and experiences of persons with chronic diseases about strategies that aim to integrate and re-integrate them into work: A systematic review of qualitative studies. *International journal of environmental research and public health*, *15*(5), 1022. | 24 | 16 | 8 |  |  |  |  |  |  |  |  |  |
| 31 | Fiore, G., Bertani, D. E., Marchi, M., Cardoso, G., & Galeazzi, G. M. (2021). Patient subjective experience of treatment with long-acting injectable antipsychotics: a systematic review of qualitative studies. *Jornal Brasileiro de Psiquiatria*, *70*, 68-77. | 11 | 5 | 6 |  |  |  |  |  |  |  |  |  |
| 32 | Fletcher, A., Crowe, M., Manuel, J., & Foulds, J. (2021). Comparison of patients’ and staff’s perspectives on the causes of violence and aggression in psychiatric inpatient settings: An integrative review. *Journal of Psychiatric and Mental Health Nursing*, *28*(5), 924-939. | 30 | 21 | 9 |  |  |  |  |  |  |  |  |  |
| 33 | Frostad Liaset, I., & Lorås, H. (2016). Perceived factors in return to work after acquired brain injury: A qualitative meta-synthesis. *Scandinavian Journal of Occupational Therapy*, *23*(6), 446-457. | 16 | 14 | 2 |  |  |  |  |  |  |  |  |  |
| 34 | Froud, R., Patterson, S., Eldridge, S., Seale, C., Pincus, T., Rajendran, D., Fossum, C., & Underwood, M. (2014). A systematic review and meta-synthesis of the impact of low back pain on people’s lives. *BMC musculoskeletal disorders*, *15*(1), 1-14. | 49 | 18 | 29 | 2 |  |  |  |  |  |  |  |  |
| 35 | Gardner, T., Refshauge, K., Smith, L., McAuley, J., Hübscher, M., & Goodall, S. (2017). Physiotherapists’ beliefs and attitudes influence clinical practice in chronic low back pain: a systematic review of quantitative and qualitative studies. *Journal of physiotherapy*, *63*(3), 132-143. | 5 | 3 | 2 |  |  |  |  |  |  |  |  |  |
| 36 | Glenton, C., Carlsen, B., Lewin, S., Wennekes, M. D., Winje, B. A., & Eilers, R. (2021). Healthcare workers’ perceptions and experiences of communicating with people over 50 years of age about vaccination: a qualitative evidence synthesis. *Cochrane Database of Systematic Reviews*(7). | 11 | 6 | 4 |  |  |  |  |  |  |  |  |  |
| 37 | Glenton, C., Colvin, C. J., Carlsen, B., Swartz, A., Lewin, S., Noyes, J., & Rashidian, A. (2013). Barriers and facilitators to the implementation of lay health worker programmes to improve access to maternal and child health: a qualitative evidence synthesis. *Cochrane Database of Systematic Reviews*(10). | 56 | 36 | 19 |  |  | 1 |  |  |  |  |  |  |
| 38 | Goulet, M.-H., Pariseau-Legault, P., Côté, C., Klein, A., & Crocker, A. G. (2020). Multiple stakeholders’ perspectives of involuntary treatment orders: a meta-synthesis of the qualitative evidence toward an exploratory model. *International Journal of Forensic Mental Health*, *19*(1), 18-32. | 44 | 37 | 7 |  |  |  |  |  |  |  |  |  |
| 39 | Hesketh, K. R., Lakshman, R., & van Sluijs, E. M. (2017). Barriers and facilitators to young children's physical activity and sedentary behaviour: a systematic review and synthesis of qualitative literature. *Obesity Reviews*, *18*(9), 987-1017. | 43 | 14 | 28 |  | 1 |  |  |  |  |  |  |  |
| 40 | Hoare, T., Vidgen, A., & Roberts, N. (2017). In their own words: a synthesis of the qualitative research on the experiences of adults seeking asylum. A systematic review of qualitative findings in forced migration. *Medicine, conflict and Survival*, *33*(4), 273-298. | 15 | 13 | 2 |  |  |  |  |  |  |  |  |  |
| 41 | Hopayian, K., & Notley, C. (2014). A systematic review of low back pain and sciatica patients' expectations and experiences of health care. *The Spine Journal*, *14*(8), 1769-1780. | 28 | 13 | 13 |  |  | 1 |  |  |  | 1 |  |  |
| 42 | Houghton, C., Dowling, M., Meskell, P., Hunter, A., Gardner, H., Conway, A., Treweek, S., Sutcliffe, K., Noyes, J., & Devane, D. (2020). Factors that impact on recruitment to randomised trials in health care: a qualitative evidence synthesis. *Cochrane Database of Systematic Reviews*(10). | 30 | 2 | 22 | 1 | 1 |  | 1 | 1 | 1 | 1 |  |  |
| 43 | Houghton, C., Meskell, P., Delaney, H., Smalle, M., Glenton, C., Booth, A., Chan, X. H. S., Devane, D., & Biesty, L. M. (2020). Barriers and facilitators to healthcare workers’ adherence with infection prevention and control (IPC) guidelines for respiratory infectious diseases: a rapid qualitative evidence synthesis. *Cochrane Database of Systematic Reviews*, *2020*(8). | 37 | 24 | 13 |  |  |  |  |  |  |  |  |  |
| 44 | Jacobsen Jardim, P. S., Hestevik, C. H., & Nøkleby, H. (2023). Co-terapi og reflekterende team i par-og familieterapi: en «mixed methods» systematisk oversikt. | 17 | 11 | 6 |  |  |  |  |  |  |  |  |  |
| 45 | Jordan, J., Rose, L., Dainty, K. N., Noyes, J., & Blackwood, B. (2016). Factors that impact on the use of mechanical ventilation weaning protocols in critically ill adults and children: a qualitative evidence‐synthesis. *Cochrane Database of Systematic Reviews*(10). | 11 | 7 | 4 |  |  |  |  |  |  |  |  |  |
| 46 | Karimi‐Shahanjarini, A., Shakibazadeh, E., Rashidian, A., Hajimiri, K., Glenton, C., Noyes, J., Lewin, S., Laurant, M., & Colvin, C. J. (2019). Barriers and facilitators to the implementation of doctor‐nurse substitution strategies in primary care: a qualitative evidence synthesis. *Cochrane Database of Systematic Reviews*(4). | 69 | 46 | 20 | 2 |  | 1 |  |  |  |  |  |  |
| 47 | Kasper, A., Mohwinkel, L.-M., Nowak, A. C., & Kolip, P. (2022). Maternal health care for refugee women-A qualitative review. *Midwifery*, *104*, 103157. | 16 | 12 | 4 |  |  |  |  |  |  |  |  |  |
| 48 | Kraft, M., & Cornelius-White, J. (2020). Adolescent experiences in wilderness therapy: A systematic review of qualitative studies. *Journal of Creativity in Mental Health*, *15*(3), 343-352. | 9 | 2 | 7 |  |  |  |  |  |  |  |  |  |
| 49 | Lewis, N. V., Feder, G. S., Howarth, E., Szilassy, E., McTavish, J. R., MacMillan, H. L., & Wathen, N. (2018). Identification and initial response to children’s exposure to intimate partner violence: a qualitative synthesis of the perspectives of children, mothers and professionals. *BMJ open*, *8*(4), e019761. | 16 | 9 | 7 |  |  |  |  |  |  |  |  |  |
| 50 | Liedberg, G. M., Björk, M., Dragioti, E., & Turesson, C. (2021). Qualitative evidence from studies of interventions aimed at return to work and staying at work for persons with chronic musculoskeletal pain. *Journal of Clinical Medicine*, *10*(6), 1247. | 18 | 9 |  |  |  |  |  |  |  |  |  |  |
| 51 | MacEachen, E., Clarke, J., Franche, R.-L., Irvin, E., & Group, W.-b. R. t. W. L. R. (2006). Systematic review of the qualitative literature on return to work after injury. *Scandinavian journal of work, environment & health*, 257-269. | 13 | 10 | 3 |  |  |  |  |  |  |  |  |  |
| 52 | MacNeela, P., Doyle, C., O'Gorman, D., Ruane, N., & McGuire, B. E. (2015). Experiences of chronic low back pain: a meta-ethnography of qualitative research. *Health psychology review*, *9*(1), 63-82. | 37 | 16 | 21 |  |  |  |  |  |  |  |  |  |
| 53 | McCulloch, S., Robertson, D., & Kirkpatrick, P. (2016). Sustaining people with dementia or mild cognitive impairment in employment: A systematic review of qualitative evidence. *British Journal of Occupational Therapy*, *79*(11), 682-692. | 8 | 7 | 1 |  |  |  |  |  |  |  |  |  |
| 54 | Megnin-Viggars, O., Symington, I., Howard, L. M., & Pilling, S. (2015). Experience of care for mental health problems in the antenatal or postnatal period for women in the UK: a systematic review and meta-synthesis of qualitative research. *Archives of women's mental health*, *18*, 745-759. | 41 | 27 | 12 |  |  |  | 2 |  |  |  |  |  |
| 55 | Merner, B., Schonfeld, L., Virgona, A., Lowe, D., Walsh, L., Wardrope, C., Graham-Wisener, L., Xafis, V., Colombo, C., & Refahi, N. (2023). Consumers’ and health providers’ views and perceptions of partnering to improve health services design, delivery and evaluation: a co‐produced qualitative evidence synthesis. *Cochrane Database of Systematic Reviews*(3). | 182 | 143 | 37 |  |  | 1 | 1 |  |  |  |  |  |
| 56 | Merry, L., Pelaez, S., & Edwards, N. C. (2017). Refugees, asylum-seekers and undocumented migrants and the experience of parenthood: a synthesis of the qualitative literature. *Globalization and health*, *13*(1), 1-17. | 138 | 108 | 29 |  |  |  |  |  |  |  |  |  |
| 57 | Mestdagh, A., & Hansen, B. (2014). Stigma in patients with schizophrenia receiving community mental health care: a review of qualitative studies. *Social psychiatry and psychiatric epidemiology*, *49*, 79-87. | 18 | 12 | 6 |  |  |  |  |  |  |  |  |  |
| 58 | Moen, Ø. L., Opheim, E., & Trollvik, A. (2019). Parents experiences raising a child with food allergy; a qualitative review. *Journal of Pediatric Nursing*, *46*, e52-e63. | 24 | 16 | 8 |  |  |  |  |  |  |  |  |  |
| 59 | Muir‐Cochrane, E., & Oster, C. (2021). Chemical restraint: a qualitative synthesis review of adult service user and staff experiences in mental health settings. *Nursing & Health Sciences*, *23*(2), 325-336. | 13 | 10 | 3 |  |  |  |  |  |  |  |  |  |
| 60 | Munabi‐Babigumira, S., Glenton, C., Lewin, S., Fretheim, A., & Nabudere, H. (2017). Factors that influence the provision of intrapartum and postnatal care by skilled birth attendants in low‐and middle‐income countries: a qualitative evidence synthesis. *Cochrane Database of Systematic Reviews*(11). | 31 | 26 | 5 |  |  |  |  |  |  |  |  |  |
| 61 | Murphy, D., Irfan, N., Barnett, H., Castledine, E., & Enescu, L. (2018). A systematic review and meta‐synthesis of qualitative research into mandatory personal psychotherapy during training. *Counselling and Psychotherapy Research*, *18*(2), 199-214. | 16 | 7 | 9 |  |  |  |  |  |  |  |  |  |
| 62 | Nematy, A., Namer, Y., & Razum, O. (2023). Lgbtqi+ refugees’ and asylum seekers’ mental health: A qualitative systematic review. *Sexuality Research and Social Policy*, *20*(2), 636-663. | 23 | 19 | 4 |  |  |  |  |  |  |  |  |  |
| 63 | Odendaal, W. A., Watkins, J. A., Leon, N., Goudge, J., Griffiths, F., Tomlinson, M., & Daniels, K. (2020). Health workers’ perceptions and experiences of using mHealth technologies to deliver primary healthcare services: a qualitative evidence synthesis. *Cochrane Database of Systematic Reviews*(3). | 53 | 26 | 23 | 3 |  |  |  |  |  | 1 |  |  |
| 64 | Pariseau-Legault, P., Vallée-Ouimet, S., Jacob, J.-D., & Goulet, M.-H. (2020). Intégration des droits humains dans la pratique du personnel infirmier faisant usage de coercition en santé mentale: recension systématique des écrits et méta-ethnographie. *Recherche en soins infirmiers*(3), 53-76. | 46 | 36 | 10 |  |  |  |  |  |  |  |  |  |
| 65 | Parsons, S., Harding, G., Breen, A., Foster, N., Pincus, T., Vogel, S., & Underwood, M. (2007). The influence of patients' and primary care practitioners' beliefs and expectations about chronic musculoskeletal pain on the process of care: a systematic review of qualitative studies. *The Clinical journal of pain*, *23*(1), 91-98. | 15 | 9 | 6 |  |  |  |  |  |  |  |  |  |
| 66 | Plahouras, J. E., Mehta, S., Buchman, D. Z., Foussias, G., Daskalakis, Z. J., & Blumberger, D. M. (2020). Experiences with legally mandated treatment in patients with schizophrenia: A systematic review of qualitative studies. *European Psychiatry*, *63*(1), e39. | 18 | 15 | 3 |  |  |  |  |  |  |  |  |  |
| 67 | Seed, T., Fox, J. R., & Berry, K. (2016). The experience of involuntary detention in acute psychiatric care. A review and synthesis of qualitative studies. *International journal of nursing studies*, *61*, 82-94. | 15 | 11 | 4 |  |  |  |  |  |  |  |  |  |
| 68 | Sim, J., & Madden, S. (2008). Illness experience in fibromyalgia syndrome: a metasynthesis of qualitative studies. *Social science & medicine*, *67*(1), 57-67. | 27 | 11 | 16 |  |  |  |  |  |  |  |  |  |
| 69 | Souza, P. P., Salata Romão, A., Rosa-e-Silva, J. C., Candido dos Reis, F., Nogueira, A. A., & Poli-Neto, O. B. (2011). Qualitative research as the basis for a biopsychosocial approach to women with chronic pelvic pain. *Journal of Psychosomatic Obstetrics & Gynecology*, *32*(4), 165-172. | 7 | 4 | 3 |  |  |  |  |  |  |  |  |  |
| 70 | Stuart, R., Akther, S. F., Machin, K., Persaud, K., Simpson, A., Johnson, S., & Oram, S. (2020). Carers' experiences of involuntary admission under mental health legislation: systematic review and qualitative meta-synthesis. *BJPsych Open*, *6*(2), e19. | 23 | 14 | 9 |  |  |  |  |  |  |  |  |  |
| 71 | Sugiura, K., Pertega, E., & Holmberg, C. (2020). Experiences of involuntary psychiatric admission decision-making: a systematic review and meta-synthesis of the perspectives of service users, informal carers, and professionals. *International journal of law and psychiatry*, *73*, 101645. | 34 | 27 | 7 |  |  |  |  |  |  |  |  |  |
| 72 | Suh, H.-W., Yoon, S.-I., Hong, S., Lee, H. W., Lee, M., Kim, J. W., & Chung, S.-Y. (2022). How do children with Tourette’s syndrome and their caregivers live with the disorder? A systematic review of qualitative evidence. *Frontiers in Psychiatry*, *13*, 992905. | 8 | 5 | 2 | 1 |  |  |  |  |  |  |  |  |
| 73 | Taylor, M., Thomas, R., Oliver, S., & Garner, P. (2022). Community views on mass drug administration for filariasis: a qualitative evidence synthesis. *Cochrane Database of Systematic Reviews*(2). | 32 | 25 | 7 |  |  |  |  |  |  |  |  |  |
| 74 | Toye, F., Pearl, J., Vincent, K., & Barker, K. (2020). A qualitative evidence synthesis using meta-ethnography to understand the experience of living with pelvic organ prolapse. *International Urogynecology Journal*, *31*, 2631-2644. | 37 | 23 | 14 |  |  |  |  |  |  |  |  |  |
| 75 | Toye, F., Seers, K., Allcock, N., Briggs, M., Carr, E., Andrews, J., & Barker, K. (2013). Patients’ experiences of chronic non-malignant musculoskeletal pain: a qualitative systematic review. *British Journal of General Practice*, *63*(617), e829-e841. | 75 | 19 | 56 |  |  |  |  |  |  |  |  |  |
| 76 | Toye, F., Seers, K., & Barker, K. (2014). A meta‐ethnography of patients' experiences of chronic pelvic pain: struggling to construct chronic pelvic pain as ‘real’. *Journal of advanced nursing*, *70*(12), 2713-2727. | 32 | 24 | 8 |  |  |  |  |  |  |  |  |  |
| 77 | Tynewydd, I., North, S., & Rushworth, I. (2020). The experiences of mental health professionals supporting forced migrants: A qualitative systematic review. *Refuge*, *36*(1), 50-65. | 11 | 10 | 1 |  |  |  |  |  |  |  |  |  |
| 78 | Velez, M., Lugo-Agudelo, L. H., Patiño Lugo, D. F., Glenton, C., Posada, A. M., Mesa Franco, L. F., Negrini, S., Kiekens, C., & Spir Brunal, M. A. (2023). Factors that influence the provision of home‐based rehabilitation services for people needing rehabilitation: a qualitative evidence synthesis. *Cochrane Database of Systematic Reviews*, *2023*(2). | 229 | 80 | 126 | 5 | 2 | 3 | 5 | 2 | 3 | 3 |  |  |
| 79 | Wingfield, T., Kirubi, B., Viney, K., Boccia, D., & Atkins, S. (2023). Experiences of conditional and unconditional cash transfers intended for improving health outcomes and health service use: a qualitative evidence synthesis. *Cochrane Database of Systematic Reviews*(3). | 116 | 45 | 57 | 4 | 2 | 1 | 1 | 2 | 4 |  |  |  |
| 80 | Wong, W. K., & Bressington, D. T. (2022). Nurses' attitudes towards the use of physical restraint in psychiatric care: A systematic review of qualitative and quantitative studies. *Journal of Psychiatric and Mental Health Nursing*, *29*(5), 659-675. | 7 | 6 | 1 |  |  |  |  |  |  |  |  |  |
| 81 | Xyrichis, A., Iliopoulou, K., Mackintosh, N. J., Bench, S., Terblanche, M., Philippou, J., & Sandall, J. (2021). Healthcare stakeholders’ perceptions and experiences of factors affecting the implementation of critical care telemedicine (CCT): qualitative evidence synthesis. *Cochrane Database of Systematic Reviews*(2). | 13 | 7 | 6 |  |  |  |  |  |  |  |  |  |
| 82 | Zhang, H., Xie, F., Yang, B., Zhao, F., Wang, C., & Chen, X. (2022). Psychological experience of COVID-19 patients: a systematic review and qualitative meta-synthesis. *American journal of infection control*, *50*(7), 809-819. | 23 | 17 | 6 |  |  |  |  |  |  |  |  |  |
